# Supplementary material for: Rapid and Simultaneous Determination of Anabolic Andro-Genic Steroids in Livestock and Poultry Meat Using One-Step Solid-Phase Extraction Coupled with UHPLC–MS/MS
Source: Molecules. 2023 Dec 22;29(1):84. doi: 10.3390/molecules29010084 (PMC10780017; doi:10.3390/molecules29010084)
Supplement: Supplementary file 1 [file molecules-29-00084-s001.zip › molecules-2745114-supplementary.pdf]

Figure S1. The precursor and product ion spectra (qualifier and quantifier) of all AASs

1 Testosterone

Precursor ion spectra

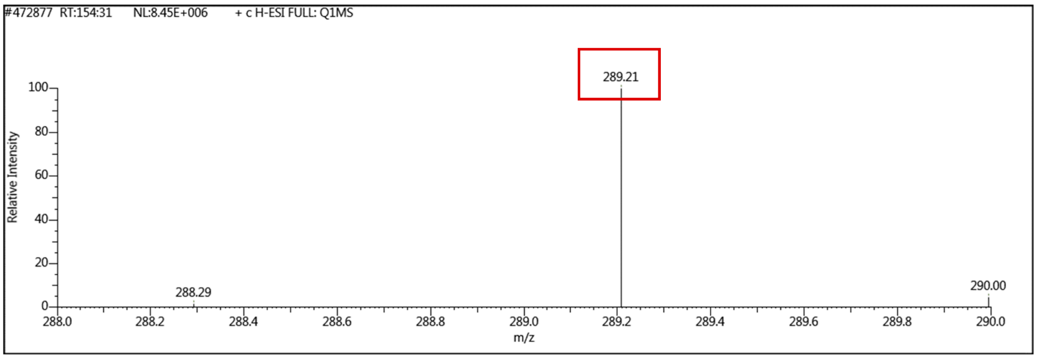

Product ion spectra

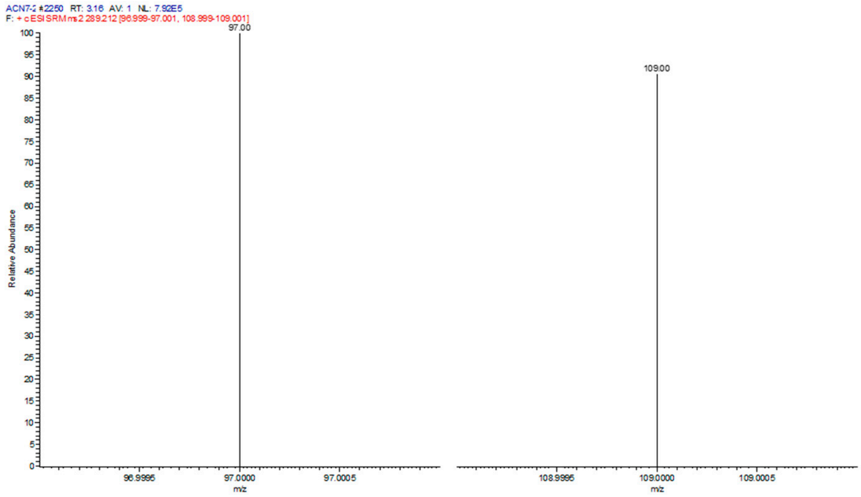

2 Epitestosterone

Precursor ion spectra

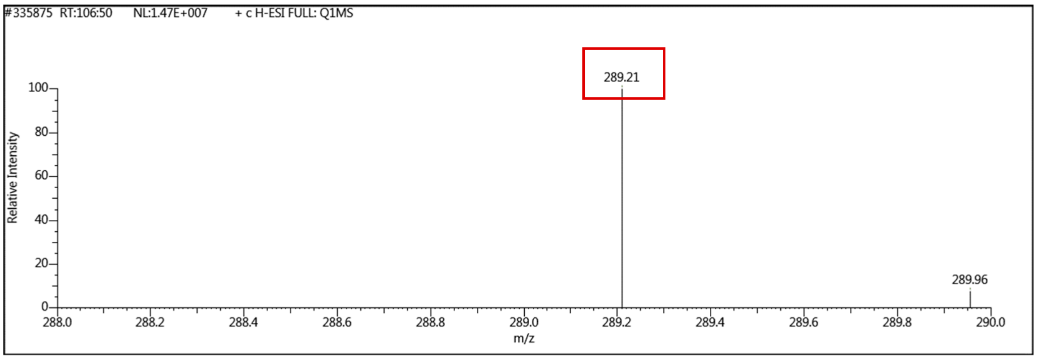

Product ion spectra

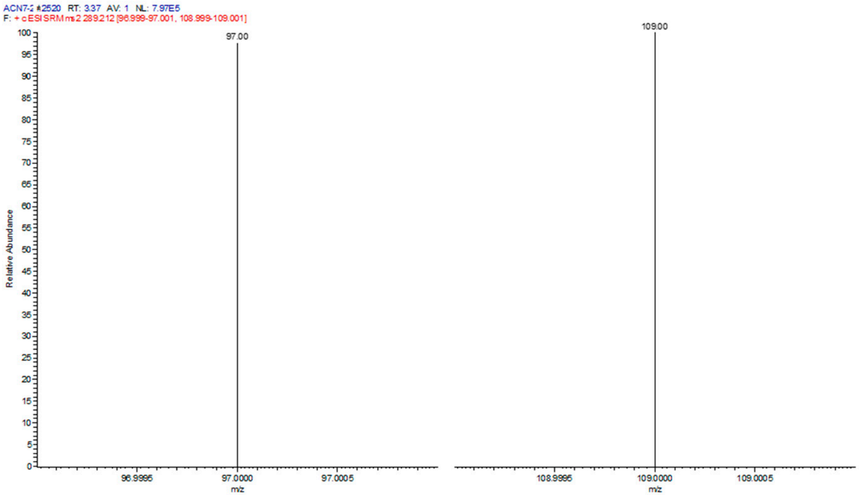

3 Methyltestosterone

Precursor ion spectra

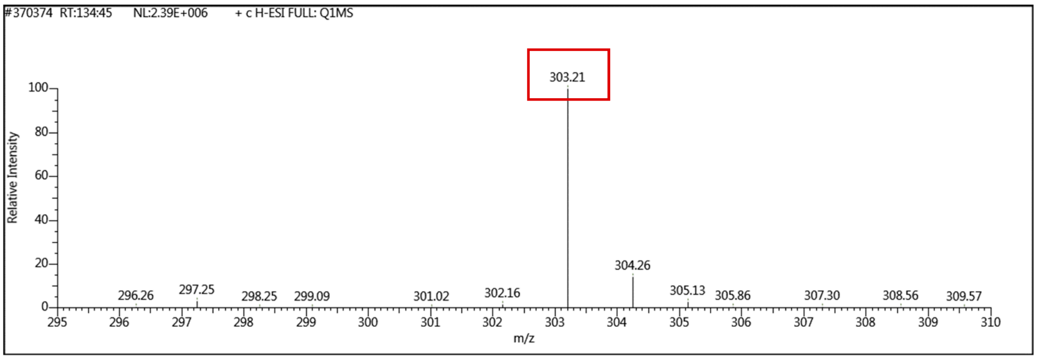

Product ion spectra

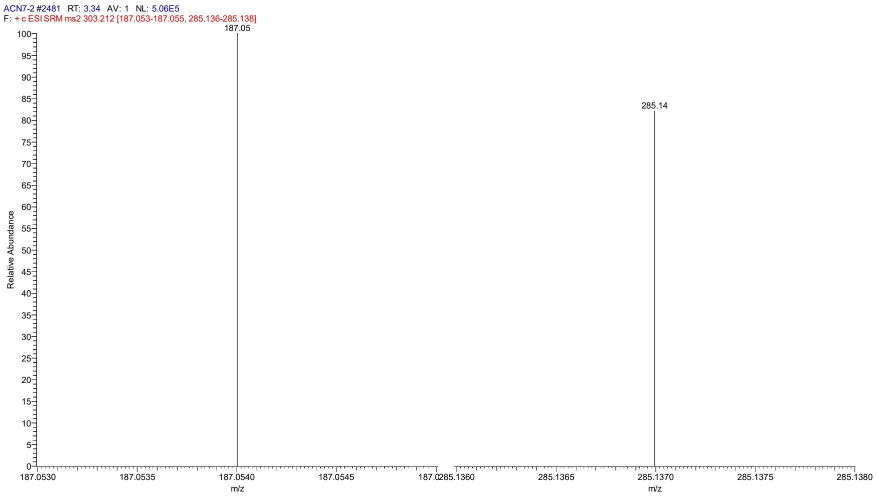

4 Nandrolone

Precursor ion spectra

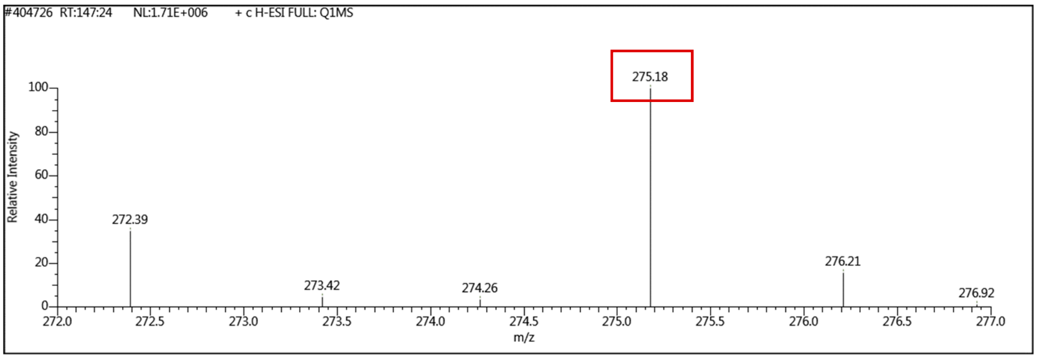

Product ion spectra

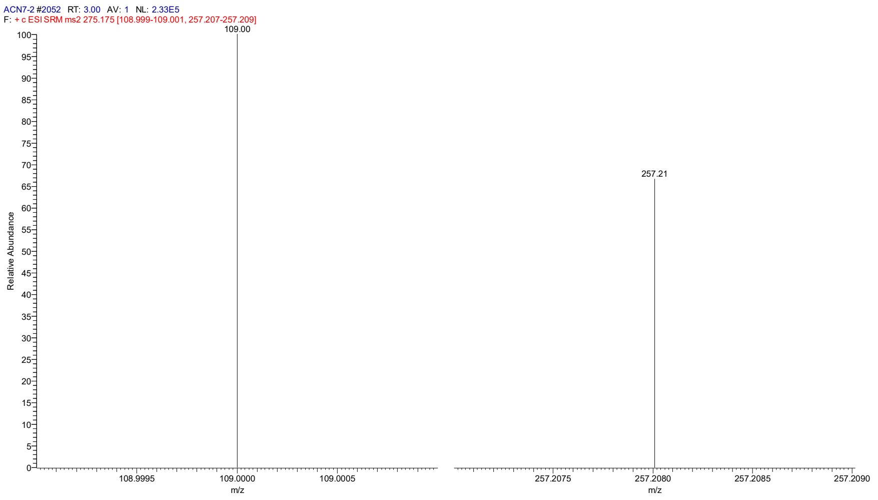

5 Boldenone

Precursor ion spectra

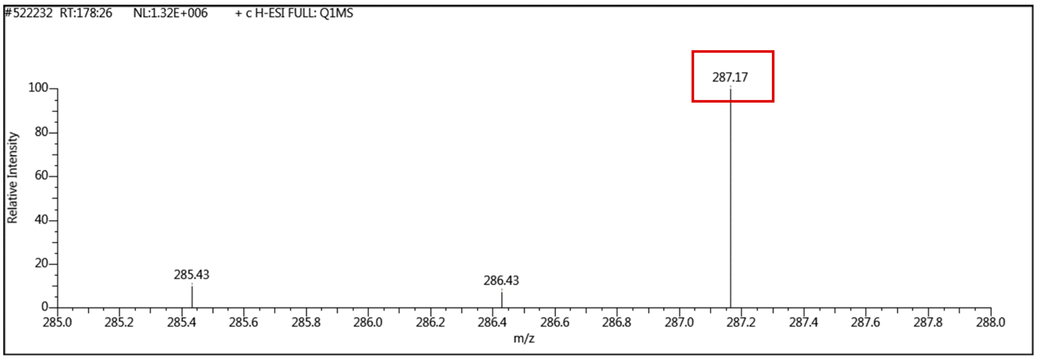

Product ion spectra

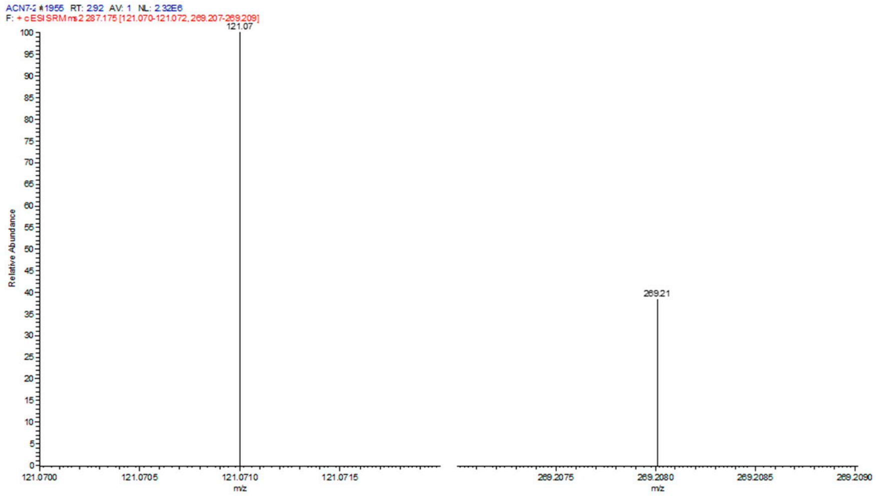

6 Metandienone

Precursor ion spectra

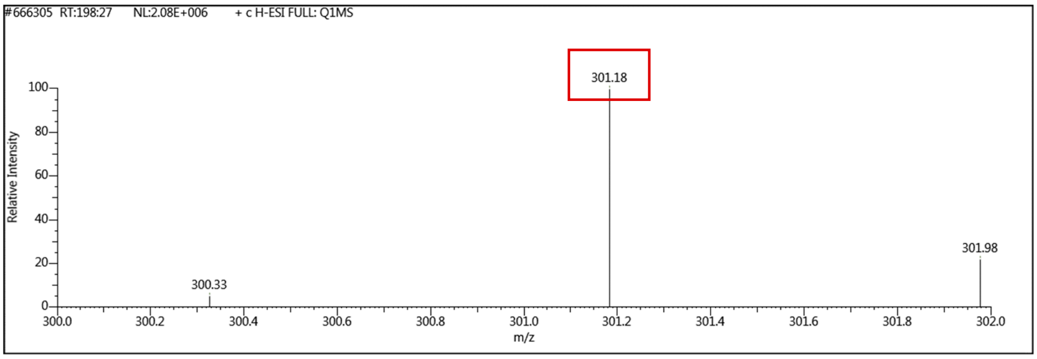

Product ion spectra

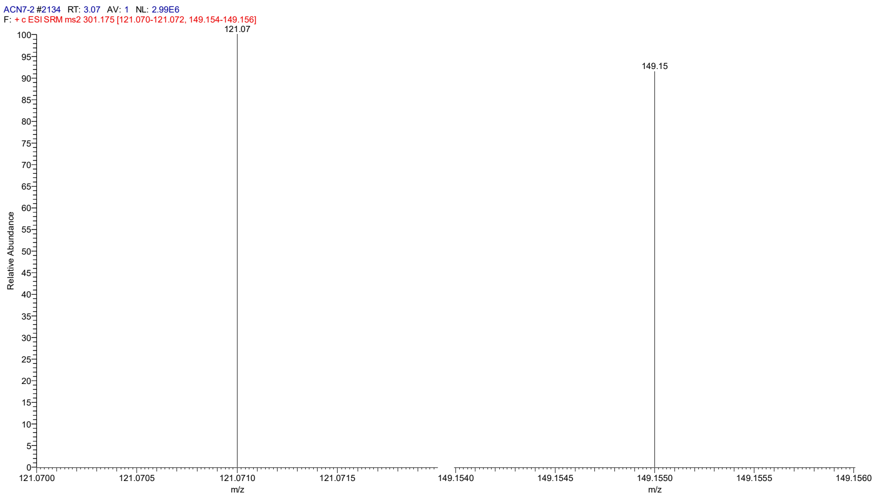

7 Trenbolone

Precursor ion spectra

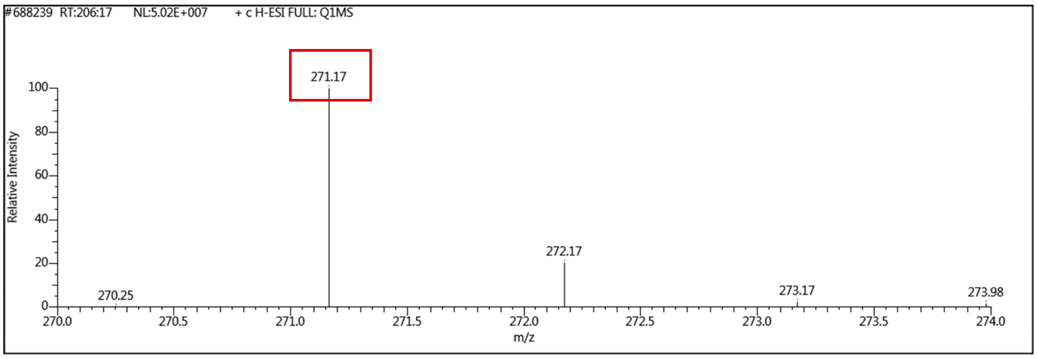

Product ion spectra

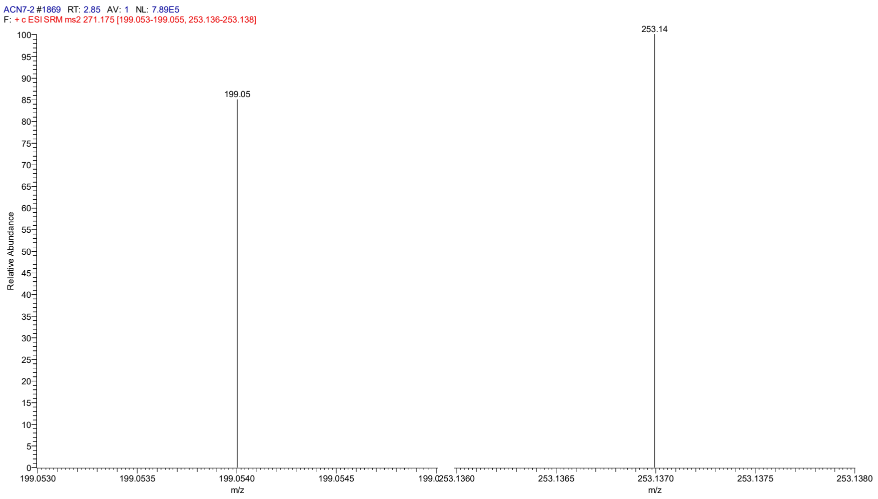

8 Metenolone

Precursor ion spectra

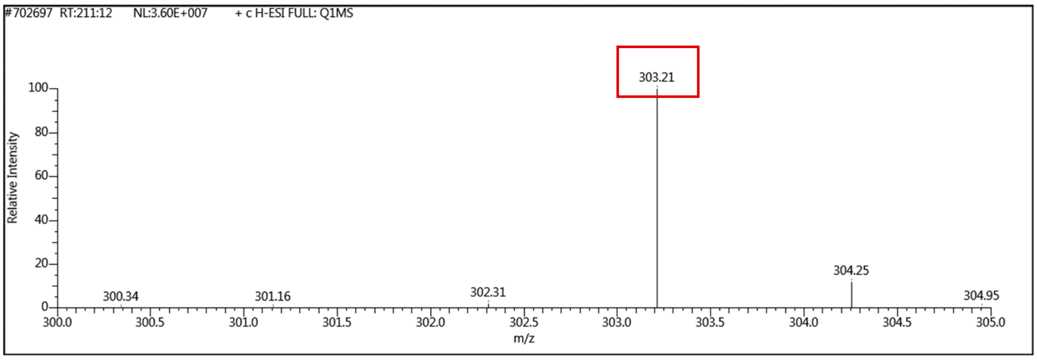

Product ion spectra

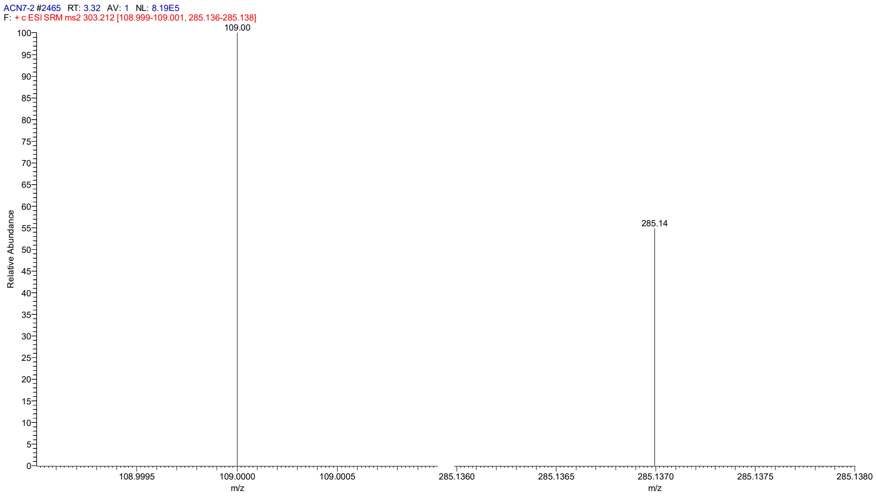

9 Methandriol

Precursor ion spectra

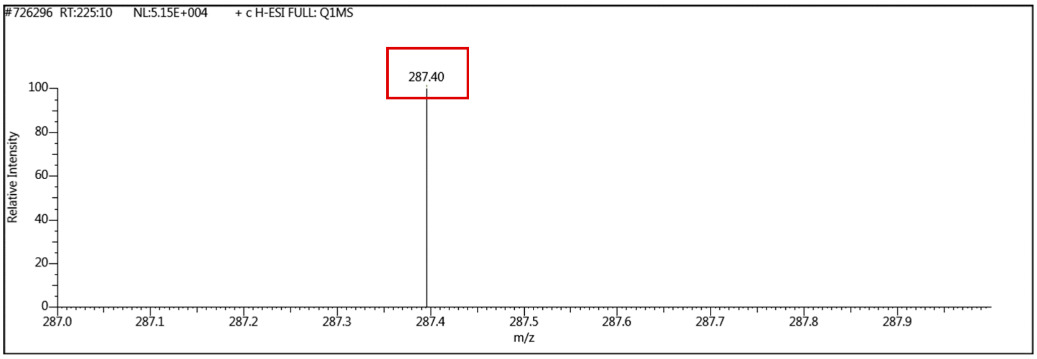

Product ion spectra

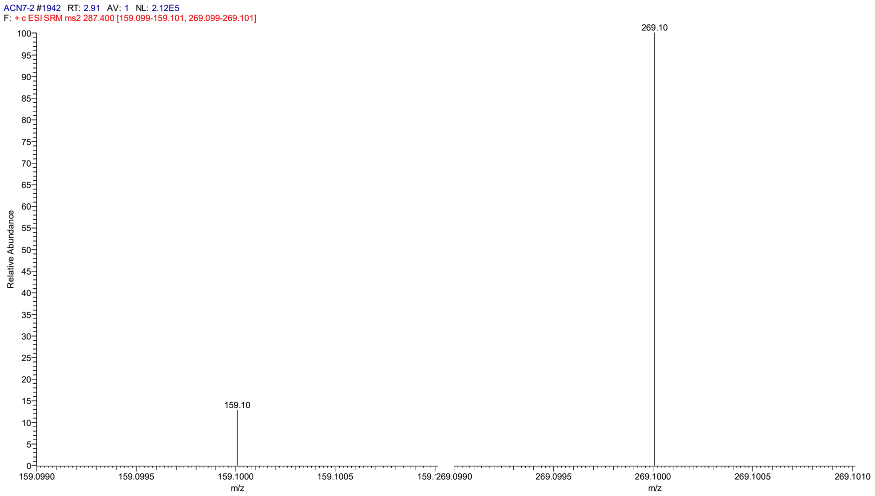

10 Mesterolone

Precursor ion spectra

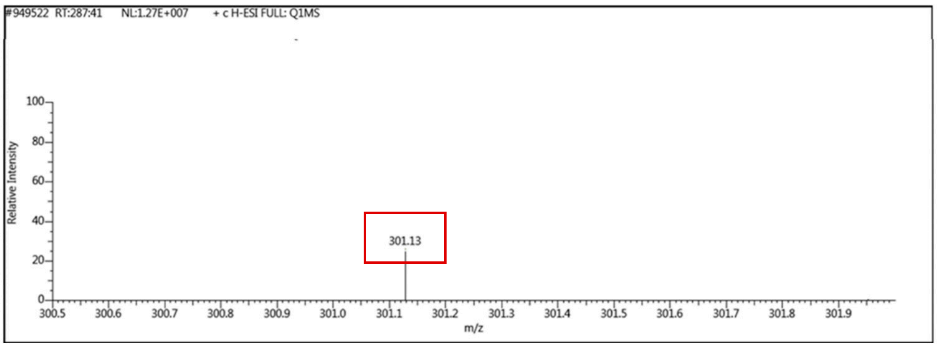

Product ion spectra

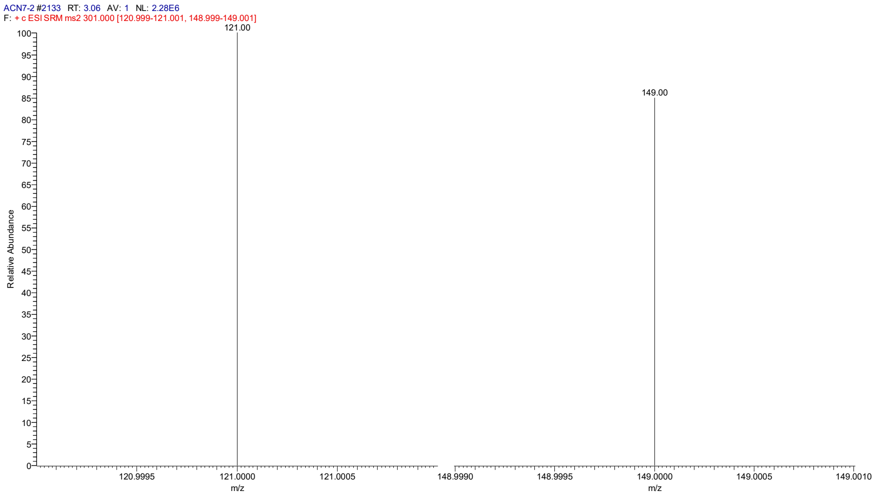

11 Danazol

Precursor ion spectra

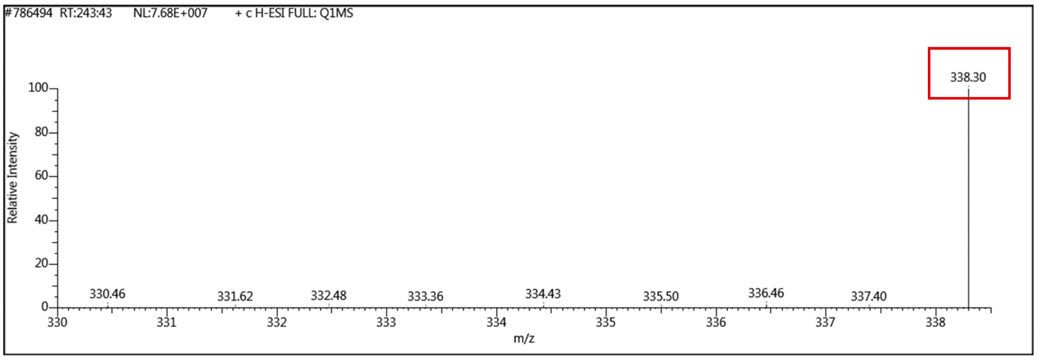

Product ion spectra

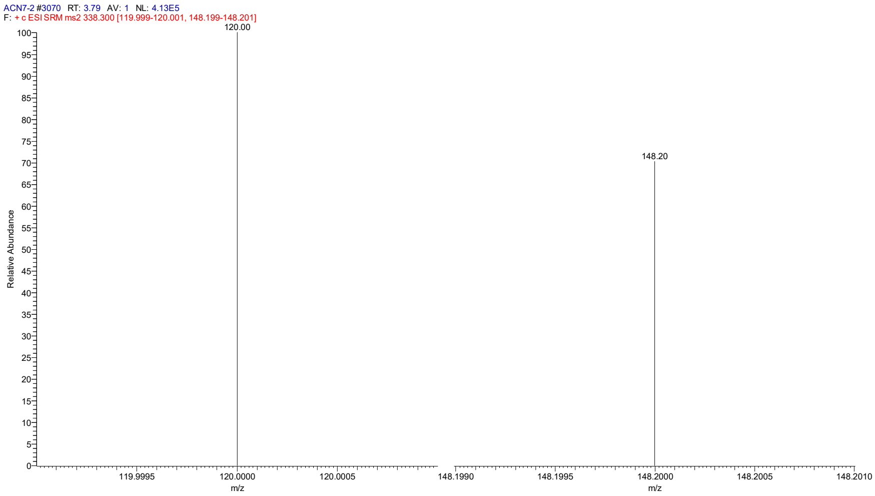

12 Stanozolol

Precursor ion spectra

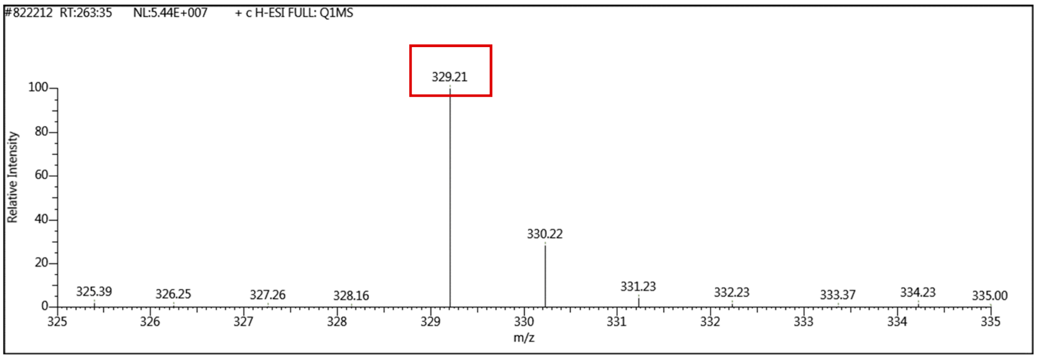

Product ion spectra

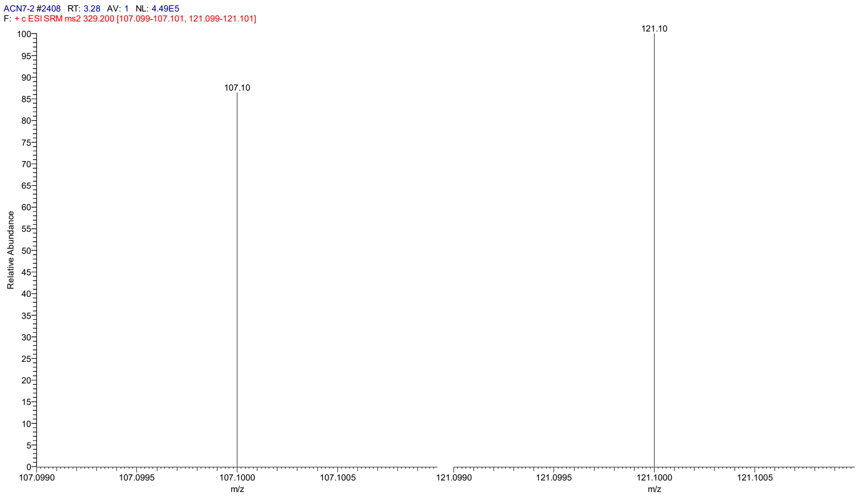
ACN7-2 #2408 RT: 3.28 AV: 1 NL: 4.49E5
F: + c ESI SRM ms2 329.200 [107.099-107.101, 121.099-121.101]
107.10
121.10
Relative Abundance
m/z
m/z
107.0990
107.0995
107.1000
107.1005
121.0990
121.0995
121.1000
121.1005
